# Modeling Chromatography Binding through Molecular Dynamics Simulations with Resin Fragments

**DOI:** 10.1021/acs.jpcb.4c00578

**Published:** 2024-05-29

**Authors:** Vitali Stanevich, Oluyemi Oyeniran, Sandeep Somani

**Affiliations:** † Protein Therapeutics API Development, Janssen Research & Development, LLC, a Johnson & Johnson company, Malvern, Pennsylvania 19355, United States; ‡ Statistics and Decision Sciences, Janssen Research & Development, LLC, a Johnson & Johnson company, Spring House, Pennsylvania 19002, United States; § In Silico Discovery, Janssen Research & Development, LLC, a Johnson & Johnson company, Spring House, Pennsylvania 19002, United States

## Abstract

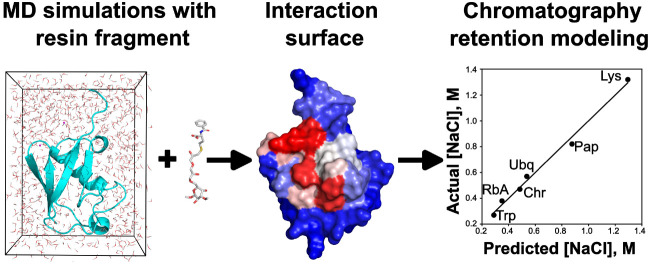

Accurate atomistic modeling of the interactions of a chromatography resin with a solute can inform the selection of purification conditions for a product, an important problem in the biotech and pharmaceutical industries. We present a molecular dynamics simulation-based approach for the qualitative prediction of interaction sites (specificity) and retention times (affinity) of a protein for a given chromatography resin. We mimicked the resin with an unrestrained ligand composed of the resin headgroup coupled with successively larger fragments of the agarose backbone. The interactions of the ligand with the protein are simulated in an explicit solvent using the Replica Exchange Molecular Dynamics enhanced sampling approach in conjunction with Hydrogen Mass Repartitioning (REMD-HMR). We computed the ligand interaction surface from the simulation trajectories and correlated the features of the interaction surface with experimentally determined retention times. The simulation and analysis protocol were first applied to a series of ubiquitin mutants for which retention times on Capto MMC resin are available. The ubiquitin simulations helped identify the optimal ligand that was used in subsequent simulations on six proteins for which Capto MMC elution times are available. For each of the six proteins, we computed the interaction surface and characterized it in terms of a range of simulation-averaged residue-level physicochemical descriptors. Modeling of the salt concentrations required for elution with respect to the descriptors resulted in a linear fit in terms of aromaphilicity and Kyte-Doolittle hydrophobicity that was robust to outliers, showed high correlation, and correctly ranked the protein elution order. The physics-based model building approach described here does not require a large experimental data set and can be readily applied to different resins and diverse biomolecules.

## Introduction

Continuous intensification and diversification of biomanufacturing processes place increasing demands on the performance of chromatography purification steps. Growing titers of upstream manufacturing increase the amount of material to be processed, requiring higher binding capacity resins. Such intensification also increases the amount of process-associated impurities, with host cell proteins (HCP)^1^ being particularly challenging. Moreover, the structural complexity of manufactured biologics is steadily growing in the form of a variety of fusion proteins, multispecific mAbs, and vaccine products. Manufacturing such elaborately engineered molecules poses special challenges due to the formation of fragments, aggregates, mis-paired conjugates, and susceptibility to post-translation modifications (PTM).^2^ In addition, critical impurities, such as low abundance HCP or PTM variants, increase the time and resource requirements of the analytical methods used for their detection, further limiting the development of chromatography processes.^3^

Multiple *in silico* approaches are being explored in the field to improve the speed, decrease costs, and increase the robustness of chromatography process development.^1^ These approaches employ different computational frameworks, such as statistical modeling based on Design of Experiment (DOE) methods, mechanistic modeling based on fundamental principles of chromatography, Quantitative Structure–Activity Relationship (QSAR) based on structural properties of purified proteins, and physics-based molecular simulations.

In this work, we employ molecular dynamics (MD) simulations to study protein–resin interactions at molecular resolution. MD simulations are routinely employed in various aspects of structure-based drug discovery, particularly to understand the interactions between ligands and receptors in solution.^4,5^ A key advantage of physics-based methods, such as MD, is that no *a priori* experimental data are required for building a predictive model. Also, mechanistic insights into protein-resin interactions can be applied to other systems and guide resin selection or composition of mobile phase for different molecules and experimental conditions.^6^

Modeling of chromatography resin at molecular resolution is challenging since the detailed composition of a chromatography resin is often not available. Resin manufacturers may provide the chemical structure of the ligand and the linker, but the structure of the polymer backbone and the location of ligand conjugation are usually not known. Moreover, in many modern chromatography resins, the ligand is not only attached to the pore wall of the base matrix but also to the polymer grafts, which further complicates the representation.^7^

The length scales of a full resin bead, including pore walls and polymeric extenders, are on the order of 50 μm, which is much longer than the typical MD system sizes, which are on the order of 100 nm. In addition, the time scales of typical MD simulations are on the order of 100 ns, which is much shorter than the experimental time scales. Due to the gap between the length and time scales of chromatography experiments and MD simulations, simplifying approximations are needed to model chromatography with MD. The following approaches have been reported in the literature: (i) free ligand that is not bound to support and at concentrations close to practically expected in a chromatographic bead;^8,9^ (ii) ligand molecules restrained to the grid mimicking a binding surface;^10−15^ (iii) a grid of ligands bound through the linker to the synthetic mimic of the agarose surface represented by Self-Assembled Monolayers (SAM) presenting chromatography ligands;^16^ (iv) ligand and linker bound to agarose helices;^17^ (v) the full chromatography bead pore with polymeric extenders.^18^ Besides variation in the components of the simulated system, different molecular resolutions have been reported (e.g., implicit solvent,^16^ coarse grained,^14,19,20^ in combination with different methods to assess the binding affinity of the protein to the resin (continuum based free energy,^16^ umbrella sampling.^14^ The methods vary in terms of the computational cost and the quantities that can be computed to benchmark against the experiments.

We present a mixed-solvent MD-based workflow to model the interaction of a protein with a known three-dimensional structure and the chromatography resin. Capto MMC resin was chosen as a model system because the molecular structure of the headgroup is known and there is substantial chromatography screening and mechanistic data using this resin. We simulated the protein with ligands representing successively larger fragments of Capto MMC resin. The ligands are untethered in the simulation box. The fragments range from a free Capto MMC headgroup to analogs with successively added linker and agarose backbone. The simulations are designed based on the hypothesis that the pattern of how a fragment “coats” the surface of the protein may correlate with chromatography observations like retention times and elution concentrations of salts. This approach is more suited for modeling nonaffinity chromatography purifications since they are nonspecific by design; therefore, the binding affinity of isolated ligands is expected to be in the low millimolar range, which may be accessible by microsecond time scale simulations employed here.

To accelerate convergence of the MD simulations, we employed the enhanced sampling approach of temperature Replica Exchange Molecular Dynamics coupled with Hydrogen Mass Repartitioning (REMD-HMR). We show that the sampling obtained from REMD-HMR simulations with a single copy of the ligand is comparable to that from extended regular MD simulations with multiple copies of the ligand. The computational cost of the simulations is roughly a week using eight GPUs.

Benchmarking the simulations against a data set of retention time and elution volumes for a series of ubiquitin mutants^21^ helped us identify the optimal ligand to represent Capto MMC resin. The ligand, named M1L1A1, contained the Capto MMC headgroup linked to one oligosaccharide unit of agarose. Using M1L1A1 as the ligand, we next simulated six other proteins for which the elution salt concentrations have been reported^22^ and computed the ligand interaction surface for each protein. The interaction surface was further characterized in terms of a range of simulation-averaged physicochemical properties of the residues constituting the interaction surface. Statistical modeling against the elution concentration for the six proteins generated a linear model in terms of aromaphilicity and hydrophobicity with a near perfect correlation. The approach described here can be readily extended to arbitrary proteins and resin chemistries.

## Methods

### Experimental Data Sets for Benchmarking

Capto MMC chromatography retention data of ubiquitin mutants were obtained from Chung et al.^21^ who reported retention times for the wild-type and 11 point mutations of ubiquitin. Another study by Holstein et al.^22^ reported the salt concentration required for protein elution from Capto MMC resin at pH 6 for six proteins, including ubiquitin. The salt concentrations range from 0.27 to 1.32 M. We used the Chung data set to develop our simulation and analysis workflow, and the Holstein data set to validate the workflow and build a model to predict elution times using simulation data.

### Molecular Dynamics Simulation Protocols

Three-dimensional structures of the proteins were obtained from the Protein Data Bank using PDB IDs 1D3Z (Ubiquitin), 1AZF (Lysozyme), 9PAP (Papain), 1CGI (α-chymotrypsinogen A), 1FS3 (Ribonuclease A), and 1AQ7 (Trypsin). All protein residues were protonated to the corresponding state at pH = 6.0 based on the p*K*_a_ determined by the APBS biomolecular solvation software suite.^23^ For all simulations, a 50 mM NaCl was used to add counterions to neutralize the simulation system.

Two types of MD simulations were performed: regular single-temperature MD and Replica Exchange MD with Hydrogen Mass Repartitioning (REMD-HMR). Both simulations used a cubic box of different sizes. In the case of regular MD, a 12 Å solvent buffer was used between the protein and the edge of the box. Box length varied between 70 Å (ubiquitin) and 80 Å (papain) depending on the protein size. For the REMD-HMR simulations, the ligand was first placed randomly around the protein such that the two were separated by 3–5 water layers, and then a cubic simulation box was set up with an extra 12 Å solvent buffer, resulting in larger box sizes. The REMD-HMR box lengths were 100 Å for ubiquitin, 110 Å for lysozyme and ribonuclease A, and 120 Å for the remaining proteins.

**Figure 1:**
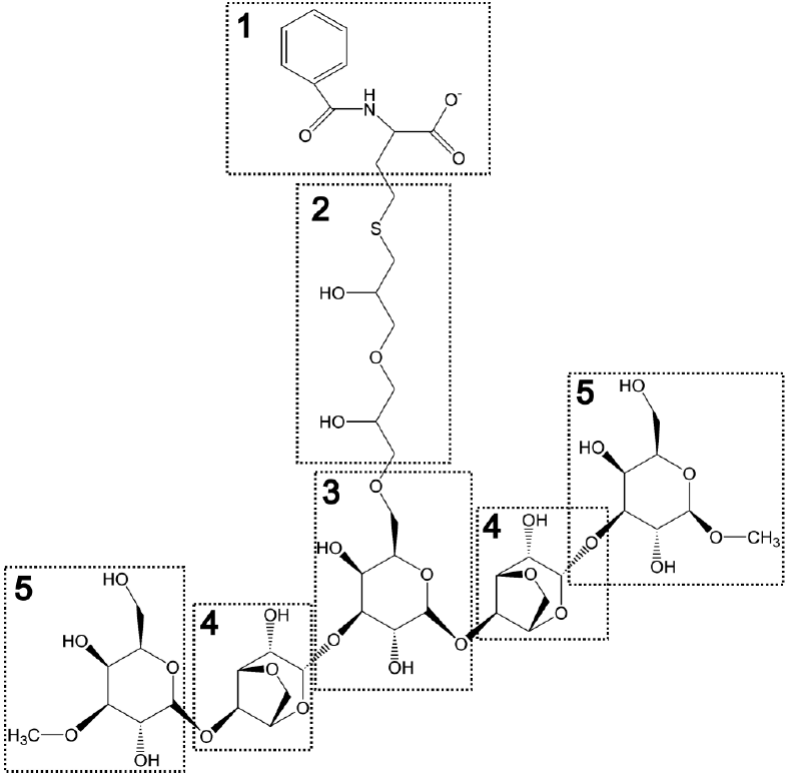
Fragments used for molecular dynamics simulations. Structures of Capto MMC resin fragments used for molecular simulations, where M1L0A0 = 1, M1L1A0 = 1 + 2, M1L1A1 = 1 + 2 + 3, M1L1A3 = 1 + 2 + 3 + 4, and M1L1A5 = 1 + 2 + 3 + 4 + 5. For all fragments only nonhydrogen atoms from block 1 were used for calculation of protein–ligand distance.

The ligands used in the simulations are specified using the following nomenclature: *M* for Capto MMC headgroup, *L* for linker, and *A* for the number of agarose residues. For example, M1L0A0 corresponds to the Capto MMC ligand without any linker or agarose residues, and M1L1A5 denotes the molecule with the Capto MMC headgroup, as well as a linker and five agarose residues (Figure 1).

Molecular fragments of Capto MMC resin were built with Avogadro software^24^ and RESP charges were derived using R.E.D. Server Development.^25^ AmberTools20 software suite^26^ was used for the assembly and parametrization of protein–ligand systems in TIP3P^27^ water with ff14SB,^28^ GLYCAM_06j-1,^29^ and GAFF^30^ force fields. The final systems were converted to GROMACS formatted coordinates and topologies with ACPYPE.^31,32^

MD simulations were performed using the GROMACS-2021.2 software.^33^ Energy was minimized by steep descent algorithm followed by equilibration in NVT and NPT ensembles with harmonic positional restraints applied to nonhydrogen atoms of protein. The temperature was maintained at 300 K with a modified Berendsen thermostat using 0.1 ps coupling time and one coupling group for the full system. Pressure was maintained with a Parrinello–Rahman barostat and 2.0 ps coupling time. Ten Å cutoffs were employed for PME electrostatics and VDW interactions. The production run for regular single-temperature MD consisted of 10 independent 2 μs unrestrained simulations, each starting from different initial velocities and a time step of 2 fs. Frames were saved every 10 ps, generating a total of 200,000 frames.

For REMD-HMR simulations, regular topology files were processed with the parmed module of AmberTools20^34^ software to increase the mass of all hydrogens by a factor of 3 while decreasing the mass of bonded heavy atoms by the same amount. The increased hydrogen mass enabled a simulation time step of 4 fs. The simulations were performed using 8 replicas with temperatures distributed between 300.0 and 308.1 K obtained by a temperature predictor for parallel tempering simulations.^35^ Each REMD replica was run for 250 ns, an aggregate simulation time of 2 μs, equivalent to a single regular MD simulation. Replica exchange was attempted every 1000 steps, and an exchange ratio of at least 10% was observed for all systems. Frames were saved every 20 ps, and only the 300 K replica was used for further analysis. Note that the temperature range of the REMD simulations here is too narrow to enhance sampling of the protein conformation, but exchanges increase the sampling of the simulation box by the ligand molecule.

### Simulation Analysis

The first step in the analysis was to compute the interaction counts of the ligand with each solvent- exposed residue of the receptor. The ligand was considered to interact with a protein residue if the distance between the center of mass of the heavy atoms of the Capto MMC headgroup (Figure 1) and any heavy atom of a protein residue was less than 4 Å. Fractional occupancy for each residue was then computed by dividing the interaction count by the total number of frames, and the interaction surface for the protein was defined as residues with fractional occupancy above 5%. The interaction surface was visualized using PyMOL 2.5.1.^36^

We then looked at the physicochemical characteristics of the residues in the interaction surface using established descriptors, such as amino acid composition and charge, aromaphilicity index,^37^ Hopp-Woods hydrophilicity index,^38^ and 11 hydrophobicity indices.^39−49^ The hydrophobicity indices reflect the variety of methods and definitions used to define the hydrophobicity of amino acids.^50^ For each descriptor, an aggregate score for the protein was obtained as the weighted average
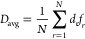
1where *d*_r_ and *f*_*r*_ are, respectively, the descriptor value and fractional occupancy for residue *r* and the sum is over the *N* of residues in the interaction surface. The descriptor values derived from the simulations were used to develop a statistical model to predict the chromatography data reported by Holstein et al.^22^

The interaction counts were also be used to compute the thermodynamics of ligand binding using the following equation:
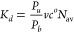
2
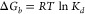
3where *K*_*d*_ is the dissociation constant, Δ*G*_*b*_ is the binding free energy, *P*_*u*_ is the fraction of unbound ligand, *P*_*b*_ is the fraction of bound ligand, *v* is volume of simulation box, *c*^0^ is standard concentration taken as 1 mol/L, *N*_av_ is Avogadro constant, *R* is the universal gas constant, and *T* is the temperature. The fraction bound and unbound are computed in terms of the fraction of frames of the full trajectory. Note that since the interactions of only the ligand headgroup are counted, not the linker or agarose backbone, the binding affinity is likely to be underestimated.

## Results

### Design of Capto MMC Mimicking Ligands

Capto MMC matrix, like many other modern chromatography resins, consists of highly cross-linked agarose, which provides a rigid and easily derivatized backbone with low nonspecific binding activity. Agarose is a natural biopolymer consisting of β-D-galactose and 3,6-anhydro-α-L-galactose monosaccharides linked by alternating β(1 → 4) and α(1 → 3) glycosidic bonds. Structurally, agarose chains assemble into double helices, which further organize into supramolecular rod structures, which then form a gel network.^51^ Derivatization of agarose by Capto MMC ligand is a nonspecific process where it can bind to any available hydroxyl group on the agarose backbone: either the primary hydroxyl group of β-D-galactose or one of the three secondary hydroxyl groups (two on β-D-galactose and one on 3,6-anhydro-α-L-galactose).

In the chromatography resin, in addition to the Capto MMC headgroup, the protein can also interact with fragments of the agarose backbone. Therefore, we performed preliminary simulations with five successively larger ligands starting from a headgroup, followed by the addition of the linker and increasing the number of agarose monosaccharides (Figure 1). The ligands were named M1L0A0, M1L1A0, M1L1A1, M1L1A3, and M1L1A5, where M1L0A0 is the smallest and contains only the headgroup, while M1L1A5 is the largest and contains linker and five agarose sugars. These simulations were used to determine the smallest ligand for which the simulations correlated to the experimental data. The resin fragment design here assumes that during derivatization, the Capto MMC ligand preferably attaches to the agarose backbone at the primary hydroxyl group of β-D-galactose, as this position is structurally more accessible.

### Application to Ubiquitin

A range of simulations were performed on wild-type ubiquitin to identify the optimal size of the Capto MMC-mimicking ligand and the appropriate MD simulation protocol. The simulations were analyzed in the context of the retention times for the 11 single-point mutants of ubiquitin reported in the Chung data set.^21^ We simulated ubiquitin with each of the five ligands shown in Figure 1 by using both regular MD and REMD-HMR simulation protocols. Following the literature precedence,^52^ we also performed simulations with a varying number of ligands in the simulation box, namely, one, three, and six. Higher copies of the ligand can speed up convergence but may also introduce unphysical ligand–ligand interactions. No repulsive forces were added between the ligands.

To assess sampling of the protein surface by the ligand, we first inspected if the ligand had nonzero interaction counts for all solvent-exposed residues. The ligand did not interact with the protein at the start of the simulations. Recall that for each of the five ligands, regular MD simulations were run with one, three, and six copies of the ligand, while REMD-HMR simulations were run only with a single ligand. All simulations except one showed nonzero interaction counts for all solvent-exposed residues (data not shown). The M1L1A5 ligand in the REMD-HMR simulation showed zero counts for 4 out of 61 surface residues. None of the 4 residues were mutated in the Chung data set.^21^

Next, we assessed the convergence of the counts for the 11 residues mutated in the Chung data set.^21^Figure S1 shows the convergence of the counts for Leu8, the residue that shows the greatest impact on the retention time upon mutation to alanine. The convergence plots show that regular MD simulations with one copy of the ligand stabilize after 10 μs and associated uncertainty does not significantly change after that. In contrast, REMD-HMR simulations stabilize within 2 μs of cumulative simulation time, in spite of 3-fold bigger simulation box by volume. After accounting for the bigger integration time step, this represents a 20-fold speed up of REMD-HMR over that of regular MD. Since the interaction counts converged for the simulations with a single copy of the ligand, subsequent simulations were performed with only a single ligand, avoiding any potential complications from ligand–ligand interactions.^53^

**Figure 2:**
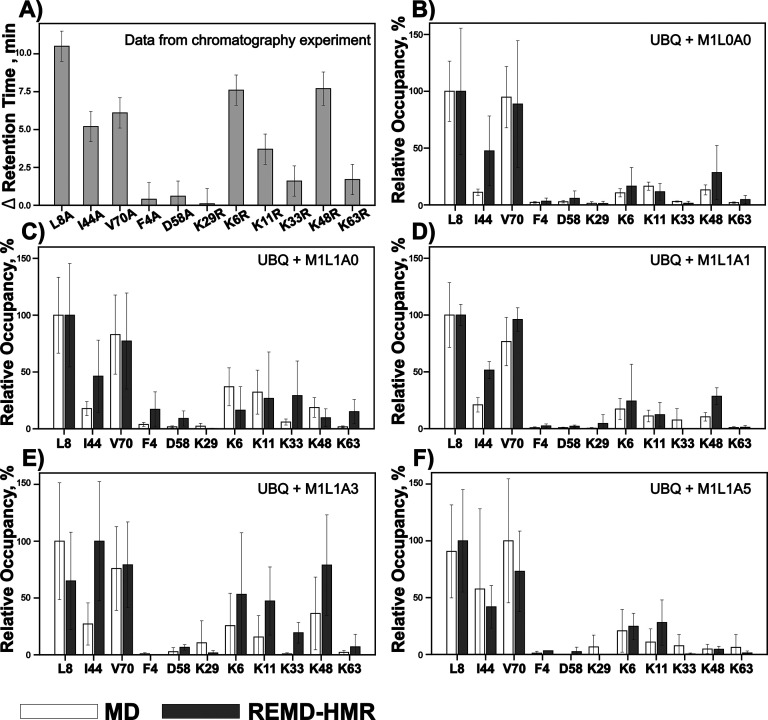
Comparison of the experimental data on the binding of ubiquitin mutants to Capto MMC resin versus binding occupancy calculated from molecular simulations with Capto MMC ligands. Readings were normalized to the highest binding residue, for the non-normalized readings, see Tables S1–4. MD is regular Molecular Dynamics with 1 copy of the ligand, REMD-HMR is Replica Exchange Molecular Dynamics with Hydrogen Mass Repartitioning. A) Experimental data plotted as absolute difference of retention time between wild-type protein and mutants, data are taken from Figure 2B of ref (21) (B–F) Molecular simulation-derived relative occupancies for Capto MMC fragments M1L0A0 (B), M1L1A0 (C), M1L1A1 (D), M1L1A3 (E), and M1L1A5 (F).

To compare the simulations with experimental retention times, we divided the mutants into three groups: (i) those with decreased (L8A, I44A and V70A); (ii) unchanged (F4A, D58A, and K29R); or (iii) increased (K6R, K48R, K11R, K33R, and K63R) retention times (Figure 2A). The classification is based on the hypothesis that the impact of the mutation on the retention time should correlate with the Capto MMC ligand occupancy of the wild-type residue. All simulations showed higher occupancy for the first group residues than for the second group residues, which is consistent with our hypothesis (Figure 2B–F, Tables S1–4). All simulations except those with the M1L1A3 ligand ranked the first group residues correctly in terms of the change in retention time: L8A > V70A > I44A. Further differentiation among the ligands comes from the analysis of the third group, which consists of lysine-to-arginine mutants increasing binding affinity. For this group, both regular MD and REMD-HMR simulations of M1L1A1 and M1L1A3 fragments best reproduced the rank ordering of the mutations with K6 and K48 being the most prominent, consistent with the experiments. K6 and K48 do not stand out as clearly for other ligands. Note that, in general, predicting the increase of retention time due to a mutation may be difficult for side chains with significantly different charge and hydrophobicity characteristics. However, lysine-to-arginine mutations are expected to increase retention times since both are positively charged but have similar physicochemical properties and lysine-to-arginine mutation is expected to increase retention time due to the arginine potential aromatic interaction side chain can also engage with the Capto MMC headgroup through aromatic interactions. Based on the above analysis, where M1L1A1 simulations have provided the best agreement with the experiment across all three mutation groups, we selected REMD-HMR simulations with the M1L1A1 ligand for subsequent simulations in this work.

**Figure 3:**
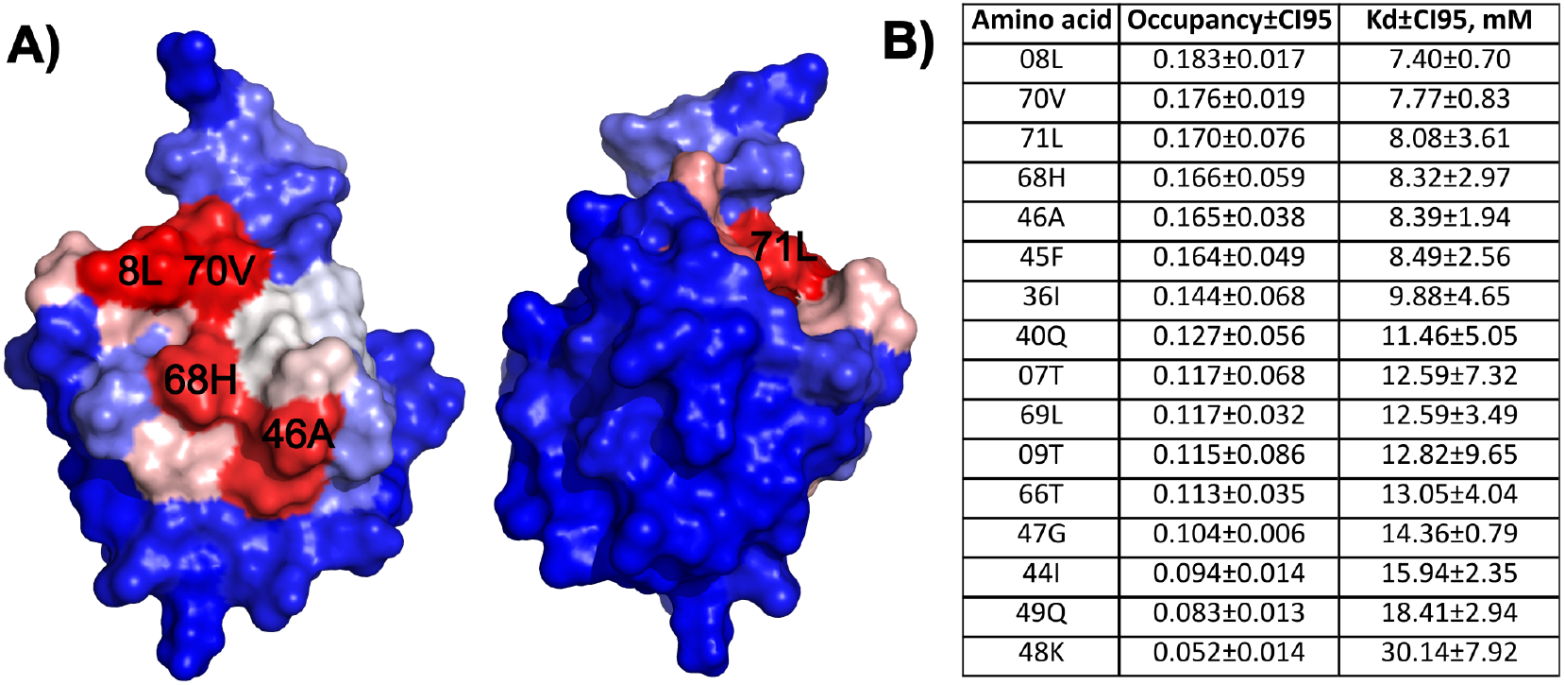
Interaction surface of ubiquitin with Capto MMC fragment M1L1A1. A) Interaction surface of ubiquitin colored in red-white-blue gradient based on ligand occupancy (red–the highest occupancy, blue–the lowest). B) Ligand occupancies and dissociation constants of top-binding ubiquitin residues.

Mapping the occupancy of the M1L1A1 ligand on the surface of ubiquitin showed a continuous interaction surface with the highest values centered at L8 and V70 (Figure 3A). The interaction surface is in good agreement with the NMR^21^ and single-molecule force spectroscopy^54^ data, further validating the current simulations. Due to the nonspecific binding, the Capto MMC ligand is expected to have multiple binding–unbinding events per simulation (Table S5), which enables straightforward calculation of binding constants based on the ratio of bound and unbound fractions of the ligand.^52,55^ Treating each residue as a binding site, we computed the dissociation constant for the M1L1A1 ligand based on its fractional occupancy and obtained affinities in the millimolar range (Figure 3B) in good agreement with the NMR data.^21^

### Building a Protein Elution Model in Terms of Simulation-Derived Quantities

**Figure 4:**
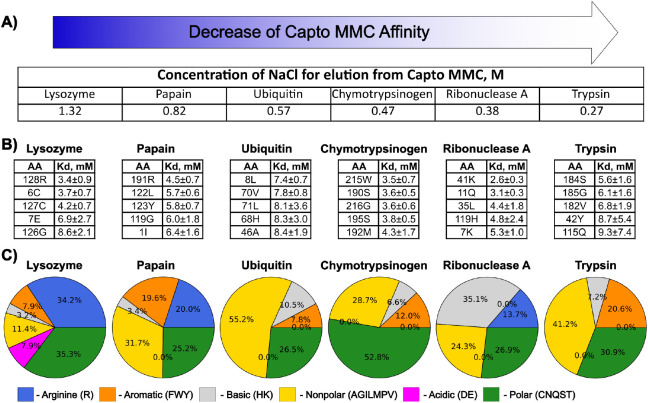
Analysis of the Capto MMC fragment M1L1A1 simulations with protein library. A) Protein affinity to Capto MMC resin as measured by gradient elution with sodium chloride. Experimental data are taken from Figure 1B of ref (22). B) Top 5 binding amino acids for each protein and their dissociation constants with a 95% confidence interval margin. C) Analysis of binding residues with occupancies >5% based on the chemical properties of the side chain.

We next performed REMD-HMR simulations with the M1L1A1 ligand on the six proteins in the Holstein data set^22^ (Figure 4A). In terms of the elution concentration of NaCl salt, the proteins ranked as lysozyme (1.32 M) > papain > ubiquitin > chymotrypsinogen > ribonuclease A > trypsin (0.27 M). For each protein, Figure S2 shows that the counts for the residue with the highest number of interactions are well converged.

**Figure 5:**
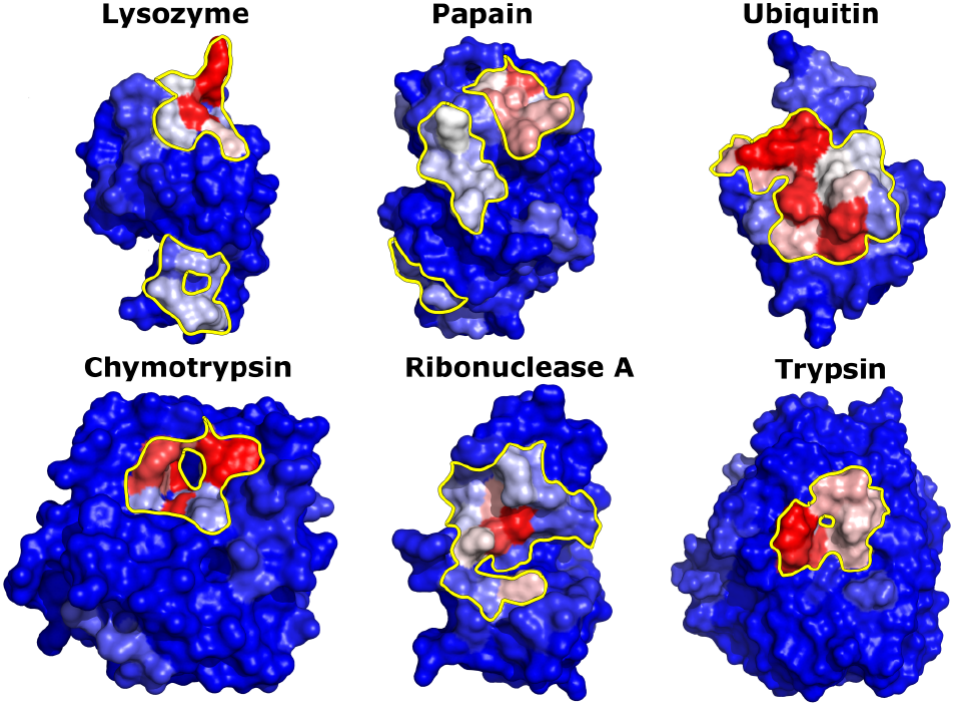
Interaction surfaces of proteins with Capto MMC fragment M1L1A1. Protein surfaces are colored in a red-white-blue gradient based on ligand occupancy (red–the highest occupancy, blue–the lowest). Residues with occupancy >5% are contoured with a yellow boundary.

As in the case of ubiquitin, we computed the occupancy of each residue and mapped it on the surface of the proteins. Figure 5 shows the residues with greater than 5% occupancy overlaid on the three-dimensional structure of the proteins. Some proteins show a single continuous patch, while others show multiple patches.

Figure 4B,C shows the amino acid composition of the interaction surface. Arginine is enriched in the proteins with the highest affinity to Capto MMC (lysozyme and papain), intermediate affinity proteins (ubiquitin and chymotrypsinogen) show hydrophobic or aromatic residues as top binders, and the weak binders (ribonuclease A and trypsin) are enriched with hydrophilic residues. However, it is not possible to rank order the proteins based on the composition of the interaction surface, suggesting that simple amino acid composition may not be sufficient to capture mixed-mode ligand binding to the resin involving both polar and nonpolar interactions.

We next computed the solvent accessible surface area (SASA) of the interaction surface (Table S6), which ranked the proteins as papain (165.8 Å^2^) > lysozyme > ribonuclease A > ubiquitin > trypsin > chymotrypsinogen (65.4 Å^2^), which was different from the ranking of the salt elution concentration. We also looked at the ranking and correlation of the salt elution concentration with respect to three more quantities computed from the simulations: total ligand occupancy (Figure S3A), highest dissociation constant (Figure S3B), and the occupancy of all arginine residues (Figure S3C). Occupancy of the arginine residues showed the highest correlation (*R*^2 ^ = 0.78) but did not rank order the proteins correctly. The other two quantities showed worse correlations and ranking.

Next, we expanded the analysis to explore if other descriptors of physicochemical properties of amino acids might be more predictive. Each amino acid in the interaction surface was assigned a value of aromaphilicity index,^37^ 11 hydrophobicity indices,^39 −49^ hydrophilicity score,^38^ and charge state based on the calculated protonation state at pH 6.0^23^. The hydrophobicity indices used here are derived from a range of hydrophobicity assessment methods which include solvent partition,^39,40^ accessible surface area,^41−43^ chromatographic,^44−46^ physical property,^47,48^ and molecular simulations.^49^ We also defined an arginine score where the arginine residues were assigned a value of 1 and the other residues were set to 0 (Table S7). The arginine score was added to the set of descriptors for subsequent statistical model building, since the arginine occupancy showed good correlation with salt elution concentration, as discussed above (Figure S3C). For each descriptor, an aggregate value that accounted for the occupancy of each residue was computed using eq 1 (Table S8).

These 15 descriptors were then used to build a statistical model to predict the salt elution concentration. When analyzed individually (see Figure S4), aromaphilicity and arginine occupancy score (AOS) showed the highest correlation (*R*^2 ^ =  0.73 for both) with the elution concentration. All other descriptors showed much poorer correlation with the Eisenberg hydrophobicity scale showing the highest correlation of *R*^2  ^=  0.38.

**Figure 6:**
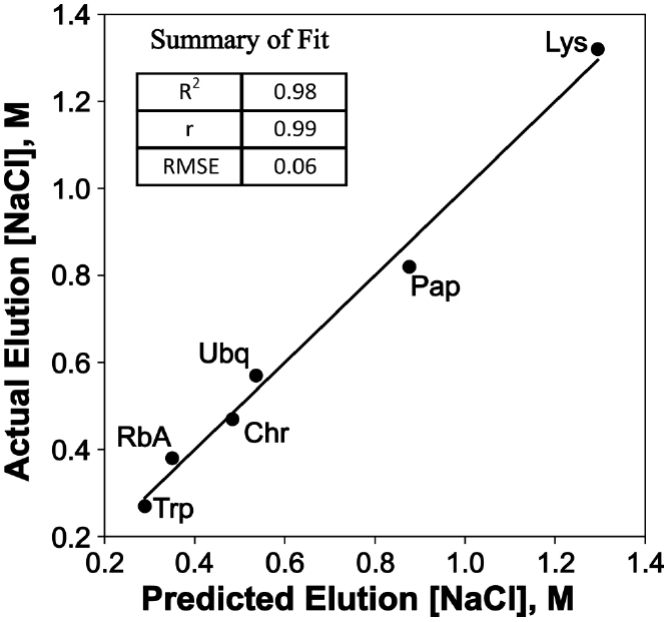
Linear fit of protein Capto MMC binding affinity versus aromaphilicity and hydrophobicity of binding site. *R*^2^ – coefficient of determination, *r*–coefficient of correlation, RMSE–Root Mean Squared Error. Lys–lysozyme, Pap–papain, Ubq–ubiquitin, Chr–chymotrypsinogen A, RbA–ribonuclease A, Trp–trypsin.

Model building was then expanded to include multiple descriptors using the stepwise selection procedure and the corrected Akaike’s Information Criterion (AICc) as the stopping criteria.^56 −59^ The accuracy of the fit was evaluated through the Root Mean Squared Error (RMSE) and coefficient of determination (*R*^2^). From the initial screening, four descriptors emerged as potential model candidates: aromaphilicity, hydrophobicity based on Kyte-Doolittle (HKD) and Radzicka-Wolfenden (HRW) scales, and hydrophilicity on Hopp-Woods scale (HHW) (Table 1). Among these descriptors, HKD and HHW are highly correlated with one another, and given the small data set including both may lead to overfitting.^60^ Indeed, the inclusion of both HKD and HHW led to a negligible change in R^2^ (by 0.014). Also, the inclusion of only HHW did not create a significant variable. Similarly, the addition of HHW and HRW only slightly increased R^2^ by 0.02, while interfering with the estimation of the impact of the residual variance. Finally, after adjusting for the small sample size, the stepwise procedure indicated aromaphilicity and HKD as the most significant variables, resulting in a linear model:
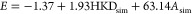
4where *E* is the predicted molar concentration of NaCl needed for elution, HKD_sim_ and *A*_sim_ are, respectively, Kyte-Doolittle hydrophobicity and aromaphilicity values derived from the simulation. This model correctly rank orders the proteins and shows a high coefficient of determination of *R*^2^ = 0.98 and low RMSE of 0.06 (Figure 6). Interestingly, the correlation of Kyte-Doolittle hydrophobicity alone is very weak (*R*^2^ = 0.10). Also, the cross-correlation between Kyte-Doolittle hydrophobicity and aromaphilicity is low, suggesting that the two descriptors capture complementary properties. It is interesting to note that the best model containing AOS was in combination with HKD (Figure S5). However, in comparison to the aromaphilicity-HKD model (*R*^2^ = 0.99, RMSE = 0.06), the AOS-HKD had a similar correlation coefficient (*R*^2^ = 0.94) but a much higher error variance (RMSE = 0.97).

**Table 1:** Results of the stepwise selection procedure with the corrected AICc as the stopping criterion for screening of computationally derived protein-MMC binding surface parameters for modeling of the NaCl concentration required for protein elution from Capto MMC column^a^

AICc	***R***^**2**^	RMSE	number of variables	DFE	variables selected
14.47	0.73	0.221	1	4	AF
27.47	0.98	0.062	2	3	AF, HKD
27.47	1.00	0.030	3	2	AF, HKD, HRW
27.47	1.00	0.002	4	1	AF, HKD, HRW, HHW

a *R*^2^–coefficient of determination, AICc–corrected Akaike’s Information Criterion, RMSE–Root Mean Squared Error, DFE–error degrees of freedom, AF– Aromaphilicity,^37^ HKD–Hydrophobicity on Kyte-Doolittle scale,^42^ HRW–Hydrophobicity on Radzicka-Wolfenden scale,^40^ HHW–Hydrophilicity on Hopp-Woods scale.^38^

The robustness of fit for the aromaphilicity-HKD model was investigated using the leave-one-out analysis where each of the proteins was sequentially omitted from the fitting data set, and the resulting model still showed high correlation and low RMSE (Table S9). Importantly, the proposed model was valid even with the omission of the proteins with the minimum and maximum binding affinity, trypsin and lysozyme, respectively. These results suggest that an analysis based on the physicochemical properties of the interaction surface residues can be more informative than that based simply on residue composition.

## Discussion

We presented a molecular dynamics simulation-based method to predict the binding of proteins to a chromatography resin. The first step of the method is to perform an all-atom mixed-solvent MD simulation to identify the residues on the surface of the protein that are likely to interact with the resin. The simulations model the resin as a fragment that represents the chemistry of both the chemical group and agarose backbone. The Capto MMC resin was used as a model system to develop the simulation and analysis workflow since extensive chromatography data are available for this resin in the literature. Based on simulations of a series of fragments, we identified the optimal fragment as the Capto MMC headgroup linked to a single residue of agarose. The results suggest that including the agarose backbone better mimics the resin compared with only the Capto MMC headgroup alone.

The simulation protocol was designed based on the hypothesis that the pattern of interaction of the resin fragment with the protein would correlate with the binding of the protein with the resin in chromatography experiments. The fragment was untethered and freely diffused in the simulation box. We developed an analysis protocol to characterize the interaction of the fragment to obtain an interaction surface from the simulations. The interaction surfaces varied widely for the six proteins simulated in this work for which chromatography purification data on Capto MMC resin have been reported before.^22^

We explored a range of increasingly complex trajectory analyses to identify descriptors of the interaction surfaces that could be used to compare the proteins and correlate the simulations with chromatography observables, such as salt elution concentrations. Based on the chemical complementarity of arginine to the Capto MMC ligand, the number of arginine residues in a protein is expected to be a potential correlate of protein binding. For the proteins simulated in this work, the number of arginine residues in the interaction surface showed a correlation with salt elution concentrations but did not accurately rank order the proteins. After evaluating a range of residue-level physicochemical descriptors, we identified Kyte-Doolittle hydrophobicity index^42^ and aromaphilicity index^37^ as significant and developed a linear model of salt elution concentrations in terms of simulation-averaged values of these descriptors for residues in the interaction surface. The model showed a near-perfect correlation and provided accurate rank ordering of the proteins. It is interesting to note that Kyte-Doolittle hydrophobicity and aromaphilicity index have a low cross-correlation, suggesting that they capture orthogonal aspects of the interaction of the protein with the resin.

One of the challenges of mixed-solvent MD simulations is achieving adequate sampling of the simulation box by the fragment. This challenge was addressed by employing temperature replica exchange, where the ligand can sample different parts of the simulation box in different replicas, and exchanges between replicas enable greater coverage of the simulation box and protein interactions. Note that replica exchange is used only for increasing the sampling of the simulation box by the fragment and not for conformational sampling of the protein. The time scales of the simulation are unlikely to capture large conformational changes, such as loop rearrangements or domain–domain motions. Further, 2-fold speed up of the simulation was achieved by using Hydrogen Mass Repartitioning. In spite of the enhanced sampling approaches used here, simulations of similar sized proteins as the systems in this work (up to 27 kDa) can take up to a week using 8 modern GPUs.

In this study, we employed the hypothesis that the impact of mutation will correlate with the interaction between ligand and wild-type protein residue. We calibrated and optimized our simulation protocol based on Capto MMC retention differences between ubiquitin mutants available in the literature.^21^ Another way to assess the impact of mutation is by performing molecular simulations on both wild-type and mutated proteins, and then correlate the difference in ligand occupancy with experimentally observed values. We plan to explore this direction in future work since such an approach will require separate simulations for each mutant, and the convergence of the differential occupancy may require longer simulations.

The experimental data set used here is admittedly small. However, we note that physics-based models typically require significantly less experimental data than machine-learning-based models. Experimental data here is only used to calibrate and benchmark the model, not to train. Nevertheless, to further validate the model, we are generating data for additional proteins, which can potentially change the model, although we expect hydrophobicity and aromaphilicity to still be significant. The variance-bias trade-off problem created in a small sample data set will also likely be resolved with a larger data set.

## Conclusion

In this article, we demonstrate a molecular dynamics simulation approach to study the specificity and binding affinity between proteins and chromatography resin. The simulations are designed to compute the residue pattern of the interaction between the protein and the resin. The simulations rely on multiple enhanced sampling algorithms and modern high-performance computing hardware to achieve sufficient convergence and statistics for further analysis.

We believe that the principles and approaches shown in this paper can find several practical implementations in the chromatographic purification of biopharmaceuticals. Mapping of the resin binding surface can be used for the assessment of the impact on resin binding of post-translational modifications, mutations, or changes of protein structure. Such knowledge can be also leveraged for the selection of resins that can most efficiently separate such protein variants. Finally, the resin binding surface from the simulations may provide insights into the binding mechanism of protein to the resin and inform the manipulation of chromatography separations through the adjustment of the mobile phase.

The method described here can guide development and save screening efforts. The workflow can be fully automated, limiting the requirement of expertise for performing the simulations and obtaining the details of the binding surface. The approach can also be readily extended to non-Capto MMC resins with different chemistries. We see a particular value of our method in studying the interaction of chromatography resin with analytically challenging solutes, such as low-abundance Host-Cell Proteins (HCPs) and Post-Translational Modifications (PTMs), in biopharmaceutical manufacturing. Application of this approach can significantly save time and money by reducing the number of chromatography experiments and the associated complex analytical testing.

Altogether, we believe that the computational approach described here can be a useful addition to the existing portfolio of *in silico* methods for understanding and influencing chromatography separations of biomolecules.

## Supporting Information Available

Additional information on protein-resin interaction sites, physicochemical descriptors of protein interaction surfaces, and correlation fits of simulation-derived quantities to chromatography data (PDF)
