# Supplementary material for: Modeling Chromatography Binding through Molecular Dynamics Simulations with Resin Fragments
Source: J Phys Chem B. 2024 May 29;128(23):5557–66. doi: 10.1021/acs.jpcb.4c00578 (PMC11181327; doi:10.1021/acs.jpcb.4c00578)
Supplement: Supplementary file 1 — jp4c00578_si_001.pdf [file jp4c00578_si_001.pdf]

Modeling Chromatography Binding through Molecular Dynamics Simulations with Resin Fragments

Vitali Stanevich[1], Oluyemi Oyeniran[2], Sandeep Somani[3]

[1] – Protein Therapeutics API Development, Janssen Research & Development, LLC, a Johnson & Johnson company, Malvern, PA, 19355, USA

[2] – Statistics and Decision Sciences, Janssen Research & Development, LLC, a Johnson & Johnson company, Spring House, PA, 19002, USA

[3] – In Silico Discovery, Janssen Research & Development, LLC, a Johnson & Johnson company, Spring House, PA, 19002, USA

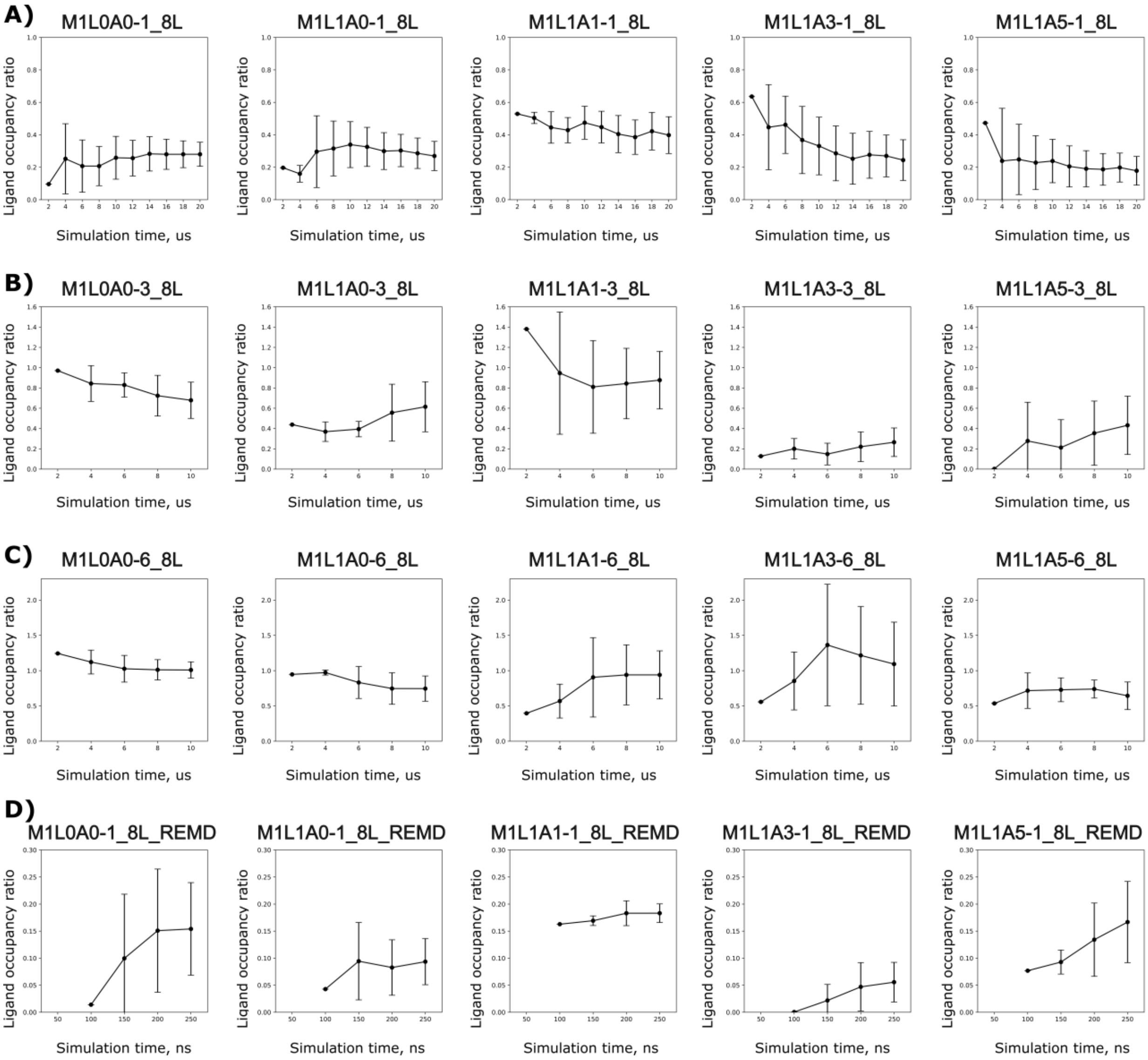

Figure S1. Dynamics of equilibration of ubiquitin Leu-8 with different screening fragments and concentrations. A) Molecular Dynamics (MD) with one molecule of MMC fragment. B) Molecular Dynamics with three molecules of MMC fragment. C) Molecular Dynamics with six molecules of MMC fragment. D) Replica Exchange Molecular Dynamics with Hydrogen Mass Repartitioning (REMD-HMR) with one molecule of MMC fragment.

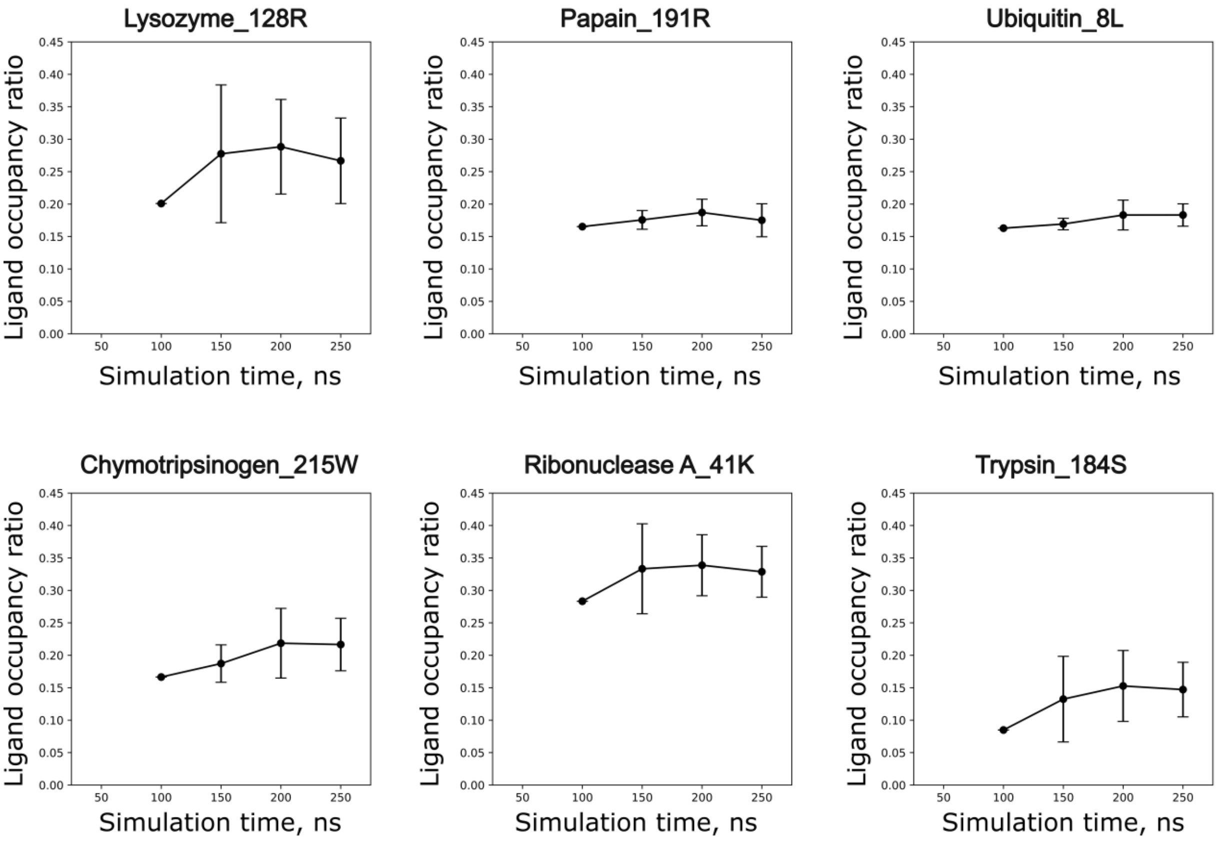

Figure S2. Convergence dynamics of top binding residue for each of the tested proteins.

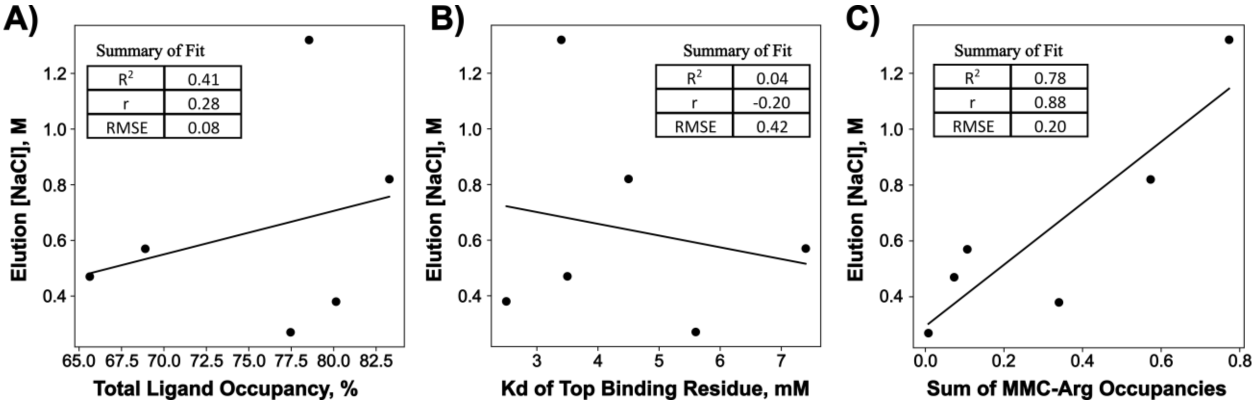

Figure S3. A) Correlation between Capto MMC binding affinity and total_protein-ligand occupancy B) Correlation between Capto MMC binding affinity and Kd of top binding residue C) Correlation of Capto MMC binding affinity and sum of Arg occupancies. R^2 – coefficient of determination, r – coefficient of correlation, RMSE – Root Mean Squared Error.

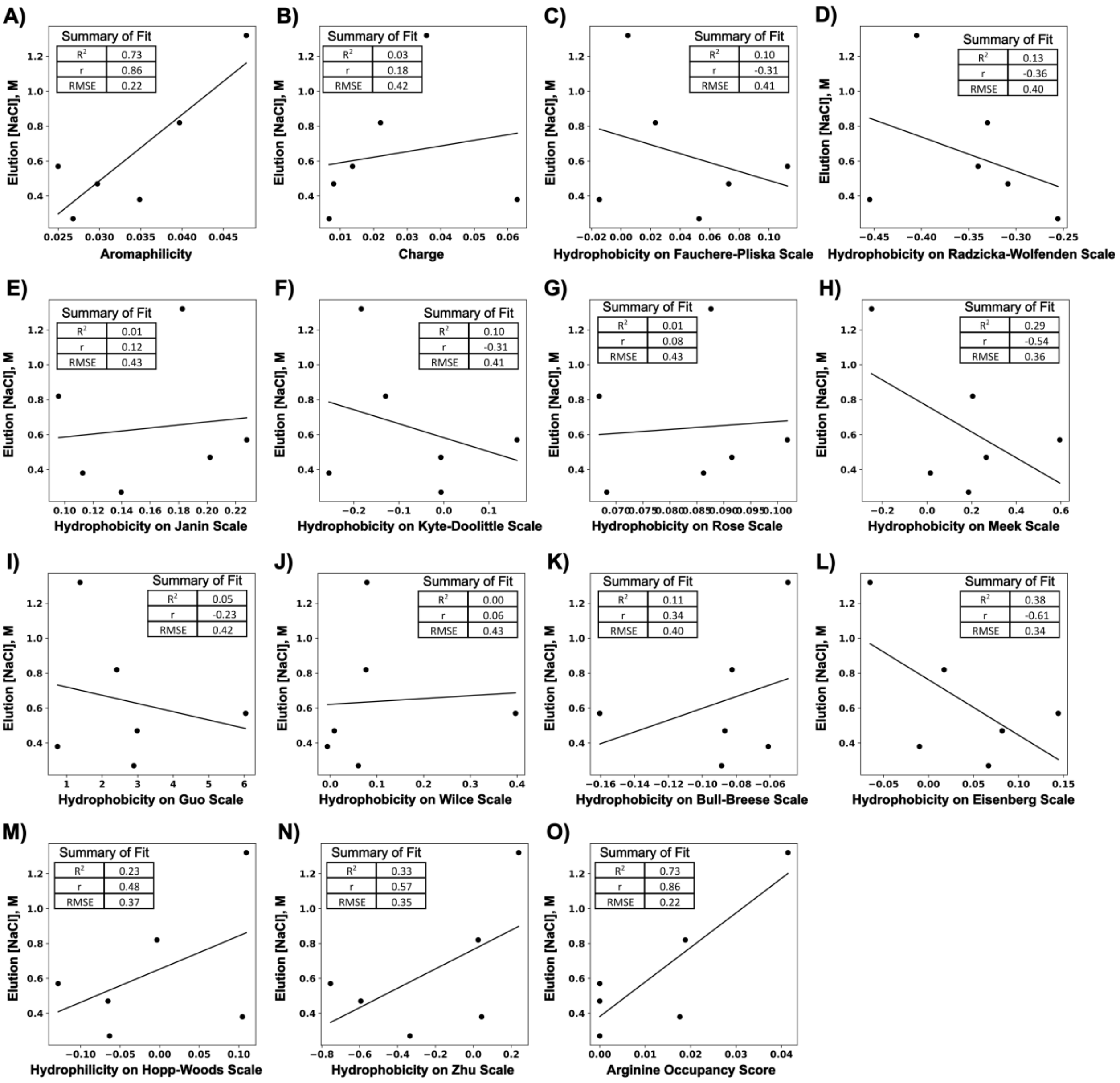

Figure S4. Correlation between molarity of NaCl required for elution from Capto MMC and property of Capto MMC binding surface derived by REMD-HMR simulations. A) Aromaphilicity [37], B) Charge at pH 6.0 [23], C) Hydrophobicity on Fauchere-Pliska scale [39], D) Hydrophobicity on Radzicka-Wolfenden scale[40], E) Hydrophobicity on Janin scale [41], F) Hydrophobicity on Kyte-Doolittle scale [42], G) Hydrophobicity on Rose scale [43], H) Hydrophobicity on Meek scale [44], I) Hydrophobicity on Guo et al scale [45], J) Hydrophobicity on Wilce et al scale [46], K) Hydrophobicity on Bull-Breese scale [47], L) Hydrophobicity on Eisenberg et al scale [48], M) Hydrophilicity on Hopp-Woods scale [38], N) Hydrophobicity on Zhu et al scale [49], O) Arginine Occupancy Score.

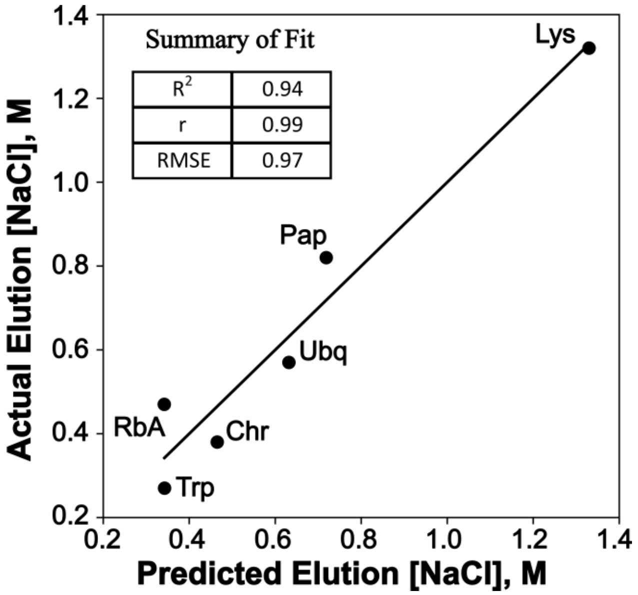

Figure S5. Linear fit of protein Capto MMC binding affinity versus Arginine Occupancy Score (AOS) and hydrophobicity as measured on Kyte-Doolittle Scale (HKD). R^2 – coefficient of determination, r – coefficient of correlation, RMSE – Root Mean Squared Error. Lys – lysozyme, Pap – papain, Ubq – ubiquitin, Chr – chymotrypsinogen A, RbA – ribonuclease A, Trp – trypsin.

Table S1. Ubiquitin-MMC binding screening by Molecular Dynamics with single MMC ligand fragments of varied complexity (M1L0A0 to M1L1A5, see main text for explanation of abbreviations). Column "Rank" represents overall rank of the residue based on ligand occupancy, column "Occ±CI95" is occupancy ± Confidence Interval of 95%. Blue, red and green colored residues are respectively hydrophobicity, charge and Lysine-to-Arginine mutants in Chung et al [21]*. Non-colored residues are nine top-binding residues other than mutated in Chung et al* [21]*.*

MD - 1 molecule

M1L0A0			M1L1A0			M1L1A1			M1L1A3			M1L1A5		
Rank	Res	Occ±CI95	Rank	Res	Occ±CI95	Rank	Res	Occ±CI95	Rank	Res	Occ±CI95	Rank	Res	Occ±CI95
1	71L	0.313±0.081	1	08L	0.269±0.090	1	08L	0.398±0.113	1	71L	0.263±0.140	1	71L	0.234±0.166
2	08L	0.280±0.074	2	71L	0.267±0.116	2	71L	0.371±0.073	2	08L	0.244±0.125	2	70V	0.218±0.119
3	70V	0.266±0.076	3	36I	0.236±0.130	3	09T	0.323±0.128	3	70V	0.185±0.090	3	08L	0.198±0.089
4	36I	0.265±0.100	4	70V	0.223±0.094	4	70V	0.305±0.085	4	69L	0.182±0.144	4	36I	0.171±0.137
5	09T	0.258±0.081	5	09T	0.200±0.087	5	07T	0.301±0.110	5	07T	0.176±0.148	5	68H	0.166±0.145
6	40Q	0.244±0.091	6	07T	0.194±0.077	6	36I	0.268±0.160	6	36I	0.171±0.156	6	09T	0.160±0.126
7	07T	0.243±0.076	7	40Q	0.188±0.104	7	69L	0.227±0.106	7	09T	0.149±0.149	7	69L	0.131±0.078
8	69L	0.207±0.072	8	69L	0.183±0.083	8	40Q	0.225±0.106	8	68H	0.144±0.085	8	44I	0.126±0.154
9	72R	0.083±0.025	9	72R	0.100±0.043	9	72R	0.168±0.078	9	40Q	0.122±0.085	9	07T	0.125±0.089
10	74R	0.079±0.030	10	06K	0.100±0.045	10	74R	0.158±0.056	10	72R	0.113±0.061	10	40Q	0.107±0.082
11	41Q	0.077±0.036	11	68H	0.098±0.041	11	42R	0.155±0.082	11	42R	0.109±0.088	11	74R	0.095±0.040
16	11K	0.046±0.010	12	11K	0.087±0.052	16	44I	0.083±0.025	14	48K	0.089±0.078	12	47G	0.084±0.076
17	48K	0.037±0.012	13	74R	0.072±0.043	17	06K	0.069±0.037	19	44I	0.066±0.045	16	06K	0.045±0.041
18	44I	0.031±0.008	19	48K	0.050±0.023	22	11K	0.044±0.020	21	06K	0.063±0.070	23	11K	0.024±0.025
19	06K	0.030±0.010	20	44I	0.048±0.017	23	48K	0.041±0.015	26	11K	0.038±0.046	28	33K	0.017±0.022
36	33K	0.008±0.001	33	33K	0.016±0.007	27	33K	0.030±0.040	30	29K	0.026±0.047	29	29K	0.014±0.023
37	58D	0.008±0.003	41	04F	0.010±0.005	48	63K	0.004±0.003	43	58D	0.007±0.009	31	63K	0.014±0.025
41	04F	0.006±0.002	48	29K	0.006±0.007	49	58D	0.003±0.002	46	63K	0.005±0.004	36	48K	0.010±0.009
42	63K	0.006±0.002	53	63K	0.005±0.002	50	04F	0.003±0.003	54	04F	0.002±0.002	53	04F	0.002±0.004
47	29K	0.004±0.004	76	58D	0.005±0.002	59	29K	0.002±0.003	56	33K	0.002±0.002	70	58D	0.000±0.000

Table S2. Ubiquitin-MMC binding screening by Molecular Dynamics with three copies of MMC ligand fragments of varied complexity (M1L0A0 to M1L1A5, see main text for meaning of abbreviations). Column "Rank" represents overall rank of the residue based on ligand occupancy, column "Occ±CI95" is occupancy ± Confidence Interval of 95%. Blue, red and green colored residues are respectively hydrophobicity, charge and Lysine-to-Arginine mutants in Chung et al [21]. *Non-colored residues are nine top-binding residues other than mutated in* Chung et al [21].

MD - 3 molecules

**M1L0A0**			**M1L1A0**			**M1L1A1**			**M1L1A3**			**M1L1A5**		
Rank	Res	Occ±CI95	Rank	Res	Occ±CI95	Rank	Res	Occ±CI95	Rank	Res	Occ±CI95	Rank	Res	Occ±CI95
1	70V	0.692±0.135	1	71L	0.779±0.183	1	08L	0.877±0.284	1	68H	0.438±0.296	1	08L	0.432±0.288
2	08L	0.678±0.179	2	08L	0.614±0.247	2	71L	0.787±0.248	2	74R	0.413±0.183	2	74R	0.368±0.372
3	71L	0.633±0.150	3	70V	0.604±0.178	3	68H	0.674±0.298	3	49Q	0.316±0.316	3	40Q	0.357±0.170
4	09T	0.566±0.203	4	09T	0.482±0.252	4	70V	0.568±0.111	4	59Y	0.281±0.229	4	71L	0.328±0.183
5	40Q	0.544±0.176	5	07T	0.456±0.259	5	72R	0.554±0.243	5	08L	0.265±0.141	5	73L	0.275±0.173
6	69L	0.541±0.162	6	72R	0.451±0.115	6	49Q	0.508±0.211	6	44I	0.253±0.180	6	72R	0.269±0.253
7	36I	0.522±0.178	7	40Q	0.447±0.224	7	09T	0.506±0.242	7	54R	0.241±0.194	7	09T	0.208±0.136
8	07T	0.486±0.136	8	74R	0.423±0.150	8	74R	0.489±0.175	8	48K	0.233±0.204	8	68H	0.166±0.167
9	41Q	0.339±0.153	9	42R	0.405±0.113	9	73L	0.454±0.202	9	47G	0.230±0.122	9	60N	0.150±0.206
10	74R	0.260±0.100	10	36I	0.398±0.269	10	07T	0.450±0.237	10	09T	0.210±0.170	10	75G	0.149±0.197
11	49Q	0.256±0.096	11	69L	0.357±0.195	11	46A	0.422±0.346	11	72R	0.205±0.179	12	58D	0.130±0.224
16	44I	0.207±0.044	15	06K	0.196±0.074	15	44I	0.336±0.093	12	46A	0.195±0.158	21	70V	0.074±0.059
17	11K	0.206±0.056	16	48K	0.154±0.032	19	06K	0.271±0.145	13	06K	0.191±0.175	24	06K	0.052±0.045
18	48K	0.200±0.046	18	44I	0.149±0.055	22	48K	0.173±0.067	18	33K	0.144±0.246	28	44I	0.043±0.036
20	06K	0.171±0.048	22	11K	0.133±0.048	28	11K	0.101±0.038	22	70V	0.108±0.037	31	63K	0.040±0.062
33	58D	0.057±0.010	36	33K	0.038±0.016	34	33K	0.060±0.044	25	11K	0.100±0.139	32	11K	0.035±0.031
39	33K	0.034±0.009	42	04F	0.022±0.007	40	63K	0.043±0.030	47	04F	0.017±0.026	34	48K	0.031±0.045
44	04F	0.027±0.007	46	63K	0.020±0.005	45	04F	0.028±0.020	50	63K	0.011±0.018	41	04F	0.012±0.020
48	63K	0.023±0.004	48	29K	0.018±0.016	48	58D	0.018±0.007	55	29K	0.007±0.013	43	33K	0.010±0.017
54	29K	0.017±0.006	76	58D	0.019±0.004	54	29K	0.012±0.015	76	58D	0.040±0.036	58	29K	0.001±0.002

Table S3. Ubiquitin-MMC binding screening by Molecular Dynamics with six copies of MMC ligand fragments of varied complexity (M1L0A0 to M1L1A5, see main text for meaning of abbreviations). Column "Rank" represents overall rank of the residue based on ligand occupancy, column "Occ±CI95" is occupancy ± Confidence Interval of 95%. Blue, red and green colored residues are respectively hydrophobicity, charge and Lysine-to-Arginine mutants in Chung et al[21]*. Non-colored residues are nine top-binding residues other than mutated in Chung et al*[21]*.*

MD - 6 molecules

**M1L0A0**			**M1L1A0**			**M1L1A1**			**M1L1A3**			**M1L1A5**		
Rank	Res	Occ±CI95	Rank	Res	Occ±CI95	Rank	Res	Occ±CI95	Rank	Res	Occ±CI95	Rank	Res	Occ±CI95
1	70V	1.064±0.058	1	71L	0.749±0.194	1	68H	1.228±0.233	1	08L	1.093±0.594	1	74R	0.769±0.420
2	08L	1.009±0.115	2	08L	0.745±0.179	2	72R	1.198±0.130	2	68H	1.007±0.193	2	68H	0.698±0.142
3	71L	0.939±0.091	3	72R	0.700±0.128	3	49Q	1.030±0.256	3	74R	0.733±0.293	3	08L	0.644±0.196
4	07T	0.774±0.101	4	70V	0.671±0.201	4	42R	1.018±0.281	4	09T	0.732±0.333	4	47G	0.623±0.254
5	69L	0.746±0.145	5	68H	0.579±0.028	5	08L	0.940±0.341	5	73L	0.699±0.477	5	71L	0.555±0.236
6	09T	0.733±0.187	6	74R	0.537±0.098	6	47G	0.779±0.056	6	71L	0.616±0.287	6	42R	0.519±0.340
7	40Q	0.680±0.185	7	42R	0.534±0.085	7	73L	0.743±0.400	7	11K	0.539±0.424	7	49Q	0.486±0.197
8	36I	0.666±0.181	8	40Q	0.504±0.146	8	46A	0.731±0.212	8	06K	0.532±0.481	8	54R	0.473±0.666
9	72R	0.508±0.159	9	07T	0.479±0.204	9	74R	0.709±0.282	9	70V	0.529±0.120	9	09T	0.453±0.260
10	49Q	0.451±0.088	10	49Q	0.470±0.096	10	48K	0.648±0.148	10	44I	0.461±0.144	10	48K	0.451±0.242
11	68H	0.440±0.095	11	09T	0.458±0.164	11	71L	0.610±0.201	11	72R	0.444±0.359	11	73L	0.411±0.299
16	44I	0.360±0.061	15	06K	0.356±0.040	12	06K	0.597±0.217	12	40Q	0.413±0.340	13	70V	0.355±0.216
18	06K	0.353±0.063	17	48K	0.281±0.064	14	70V	0.537±0.124	13	47G	0.378±0.099	14	58D	0.301±0.492
19	11K	0.331±0.052	18	44I	0.280±0.055	16	44I	0.451±0.211	14	34E	0.370±0.257	15	44I	0.263±0.084
20	48K	0.314±0.022	23	11K	0.192±0.056	22	11K	0.300±0.161	25	48K	0.096±0.062	22	06K	0.148±0.088
34	58D	0.099±0.014	40	33K	0.068±0.021	30	33K	0.115±0.074	32	33K	0.025±0.019	40	11K	0.044±0.045
37	33K	0.080±0.013	41	63K	0.067±0.019	32	04F	0.113±0.067	36	63K	0.010±0.017	45	63K	0.029±0.049
45	29K	0.059±0.024	43	58D	0.059±0.011	43	58D	0.046±0.016	40	58D	0.006±0.008	49	33K	0.005±0.009
47	04F	0.052±0.007	47	04F	0.047±0.007	46	63K	0.038±0.023	43	04F	0.005±0.004	50	04F	0.004±0.006
48	63K	0.051±0.012	60	29K	0.018±0.010	59	29K	0.010±0.013	74	29K	0.000±0.000	72	29K	0.000±0.000

Table S4. Ubiquitin-MMC binding screening by Replica Exchange Molecular Dynamics with applied Hydrogen Mass Repartitioning (REMD-HMR) with one molecule of MMC ligand fragment of varied complexity (M1L0A0 to M1L1A5, see main text for meaning of abbreviations). Column "Rank" represents overall rank of the residue based on ligand occupancy, column "Occ±CI95" is occupancy ± Confidence Interval of 95%. Blue, red and green colored residues are respectively hydrophobicity, charge and Lysine-to-Arginine mutants in Chung et al[21]. Non-colored residues are nine top-binding residues other than mutated in Chung et al[21].

REMD-HMR - 1 molecule														
M1L0A0			M1L1A0			M1L1A1			M1L1A3			M1L1A5		
Rank	Res	Occ±CI95	Rank	Res	Occ±CI95	Rank	Res	Occ±CI95	Rank	Res	Occ±CI95	Rank	Res	Occ±CI95
1	08L	0.154±0.086	1	74R	0.146±0.058	1	08L	0.183±0.017	1	42R	0.157±0.025	1	71L	0.202±0.058
2	70V	0.137±0.086	2	08L	0.094±0.043	2	70V	0.176±0.019	2	74R	0.155±0.024	2	08L	0.167±0.075
3	07T	0.133±0.079	3	73L	0.091±0.034	3	71L	0.170±0.076	3	49Q	0.106±0.019	3	68H	0.139±0.043
4	71L	0.129±0.056	4	70V	0.072±0.040	4	68H	0.166±0.059	4	44I	0.086±0.045	4	07T	0.125±0.024
5	09T	0.121±0.065	5	71L	0.058±0.039	5	46A	0.165±0.038	5	71L	0.085±0.049	5	70V	0.122±0.059
6	36I	0.113±0.066	6	45F	0.045±0.063	6	45F	0.164±0.049	6	68H	0.083±0.025	6	09T	0.120±0.045
7	69L	0.098±0.059	7	44I	0.043±0.030	7	36I	0.144±0.068	7	09T	0.074±0.039	7	47G	0.102±0.021
8	40Q	0.092±0.068	8	62Q	0.043±0.067	8	40Q	0.127±0.056	8	73L	0.074±0.044	8	73L	0.099±0.033
9	68H	0.084±0.058	9	65S	0.042±0.069	9	07T	0.117±0.068	9	75G	0.069±0.034	9	36I	0.096±0.014
10	44I	0.073±0.047	10	60N	0.040±0.061	10	69L	0.117±0.032	10	70V	0.068±0.032	10	74R	0.093±0.036
11	47G	0.062±0.037	11	72R	0.037±0.039	11	09T	0.115±0.086	11	48K	0.068±0.038	11	69L	0.092±0.017
12	49Q	0.058±0.038	12	09T	0.035±0.031	14	44I	0.094±0.014	12	47G	0.067±0.026	14	44I	0.070±0.031
14	48K	0.044±0.037	18	33K	0.027±0.028	16	48K	0.052±0.014	15	08L	0.056±0.037	19	11K	0.047±0.033
21	06K	0.025±0.025	20	11K	0.025±0.038	17	06K	0.045±0.059	17	06K	0.046±0.046	20	06K	0.041±0.019
24	11K	0.018±0.011	30	04F	0.016±0.014	23	11K	0.023±0.020	19	11K	0.041±0.026	32	48K	0.008±0.004
34	58D	0.009±0.010	31	06K	0.015±0.019	37	29K	0.008±0.014	28	33K	0.017±0.008	35	04F	0.005±0.009
36	63K	0.007±0.006	32	63K	0.014±0.010	43	04F	0.004±0.003	45	63K	0.006±0.010	38	58D	0.004±0.007
39	04F	0.005±0.004	38	48K	0.009±0.007	45	58D	0.004±0.002	46	58D	0.006±0.002	46	63K	0.002±0.003
51	33K	0.002±0.003	41	58D	0.009±0.006	48	63K	0.002±0.003	54	29K	0.001±0.002	51	33K	0.001±0.001
56	29K	0.002±0.003	61	29K	0.000±0.000	64	33K	0.000±0.000	65	04F	0.000±0.000	66	29K	0.000±0.000

Table S5. Bound ratio of ubiquitin with single copy of Capto MMC ligand.

Fragment	Simulation method	Chemical head bound ratio, %	Whole ligand bound ratio, %
M1L0A0	REMD-HMR	40.4±14.2	40.4±14.2
M1L1A0	REMD-HMR	43.9±6.7	48.6±5.2
M1L1A1	REMD-HMR	68.9±4.7	76.0±3.1
M1L1A3	REMD-HMR	67.5±11.1	91.6±9.0
M1L1A5	REMD-HMR	69.0±5.7	93.1±4.0
M1L1A1	MD, 1 ligand	90.4±3.7	96.1±2.6

Table S6. Quantification of Solvent Accessible Surface Area (SASA) of total protein and of residues with M1L1A ligand occupancy > 5%.

**Protein**	**Total SASA, Å^2**	**SASA of residues with ligand occupancy >5%, Å^2**
Lysozyme	693.5	150.8
Papain	1005.9	165.8
Ubiquitin	484.6	120.5
Chymotrypsinogen	1116.5	65.4
Ribonuclease A	749.7	141.6
Trypsin	972.0	87.4

Table S7. Amino acid index scales used in the article. AF – Aromaphilicity[37], HKD – Hydrophobicity on Kyte-Doolittle scale[42], HHW – Hydrophilicity on Hopp-Woods scale [38], HRW – Hydrophobicity on Radzicka-Wolfenden scale[40], HE – Hydrophobicity on Eisenberg scale[48], HR – Hydrophobicity on Rose scale[43], HJ – Hydrophobicity on Janin scale[41], HFP – Hydrophobicity on Fauchere-Pliska scale[39], HM – Hydrophobicity on Meek scale[44], HG – Hydrophobicity on Guo scale[45], HW – Hydrophobicity on Wilce scale[46], HBB – Hydrophobicity on Bull-Breese scale[47], HZ - Hydrophobicity on Zhu scale[49], AOS – Arginine Occupancy Score.

	**AF**	**HKD**	**HHW**	**HRW**	**HE**	**HR**	**HJ**	**HFP**	**HM**	**HG**	**HW**	**HBB**	**HZ**	**AOS**
Ala	0.025	1.8	-0.5	0.13	0.67	0.74	1.7	0.31	0.5	25	2.62	-0.2	-9.58	0
Leu	0.125	3.8	-1.8	-2.64	1.9	0.85	2.4	1.7	8.8	100	6.57	-2.46	-8.66	0
Arg	0.75	-4.5	3	-5	-2.1	0.64	0.1	-1.01	0.8	-7	1.26	-0.12	11.18	1
Lys	0.1	-3.9	3	-3.97	-0.57	0.52	0.05	-0.99	0.1	-26	-2.78	-0.35	1.71	0
Asn	0.2	-3.5	0.2	-3.04	-0.6	0.63	0.4	-0.6	0.8	-7	-1.27	0.08	2.76	0
Met	0.325	1.9	-1.3	-3.83	2.4	0.85	1.9	1.23	4.8	68	-3.12	-1.47	-7.65	0
Asp	-0.025	-3.5	3	-2.23	-1.2	0.62	0.4	-0.77	-8.2	2	-2.84	-0.2	32.72	0
Phe	0.575	2.8	-2.5	-3.74	2.3	0.88	2.2	1.79	13.2	100	9.14	-2.33	-9.23	0
Cys	0.2	2.5	-1	-2.52	0.38	0.91	4.6	1.54	-6.8	32	0.73	-0.45	-5.63	0
Pro	0.125	-1.6	0	0	1.2	0.64	0.6	0.72	6.1	25	-0.12	-0.98	-7.55	0
Gln	0.3	-3.5	0.2	-3.84	-0.22	0.62	0.3	-0.22	-4.8	0	-1.69	0.16	2.02	0
Ser	0.125	-0.8	0.3	-1.66	0.01	0.66	0.8	-0.04	1.2	-2	-1.39	-0.39	-3.56	0
Glu	0.05	-3.5	3	-3.43	-0.76	0.62	0.3	-0.64	-16.9	14	-0.45	-0.3	6.16	0
Thr	0.075	-0.7	-0.4	-2.31	0.52	0.7	0.7	0.26	2.7	7	1.81	-0.52	-4.06	0
Gly	0	-0.4	0	1.45	0	0.72	1.8	0	0	-2	-1.15	0	-4.57	0
Trp	1	-0.9	-3.4	-8.21	2.6	0.85	1.6	2.25	14.9	109	5.91	-2.01	-8.62	0
His	0.45	-3.2	-0.5	-5.61	0.64	0.78	0.8	0.13	-3.5	-26	-0.74	-0.12	1.41	0
Tyr	0.85	-1.3	-2.3	-5.97	1.6	0.76	0.5	0.96	6.1	56	1.39	-2.24	-0.05	0
Ile	0.2	4.5	-1.8	-2.77	1.9	0.88	3.1	1.8	13.9	91	4.38	-2.26	-9.73	0
Val	0.15	4.2	-1.5	-2.05	1.5	0.86	2.9	1.22	2.7	62	2.3	-1.56	-8.26	0

Table S8. Parameters used for model building of protein elution from Capto MMC resin. El_NaCl – molar concentration of NaCl in elution buffer from Capto MMC resin [22], AF – binding site aggregate aromaphilicity, HKD – binding site aggregate hydrophobicity on Kyte-Doolittle scale, HHW – binding site aggregate hydrophilicity on Hopp-Woods scale, HRW – binding site aggregate hydrophobicity on Radzicka-Wolfenden scale, HE – binding site aggregate hydrophobicity on Eisenberg scale, HR – binding site aggregate hydrophobicity on Rose scale, HJ – binding site aggregate hydrophobicity on Janin scale, HFP – binding site aggregate hydrophobicity on Fauchere-Pliska scale, HM – binding site aggregate hydrophobicity on Meek scale, HG – binding site aggregate hydrophobicity on Guo scale, HW – binding site aggregate hydrophobicity on Wilce scale, HBB – binding site aggregate hydrophobicity on Bull-Breese scale, HZ - binding site aggregate hydrophobicity on Zhu scale, CH – binding site aggregate charge calculated at pH 6.0, AOS – binding site aggregate Arginine Occupancy Score.

	El_NaCl	AF	HKD	HHW	HRW	HE	HR	HJ	HFP	HM	HG	HW	HBB	HZ	CH	AOS
Lysozyme	1.32	0.04778	-0.18366	0.10945	-0.40512	-0.06493	0.08763	0.18257	0.00468	-0.24851	1.37544	0.07854	-0.04947	0.23945	0.03578	0.04143
Papain	0.82	0.03969	-0.12935	-0.00346	-0.33004	0.01739	0.06685	0.09570	0.02320	0.20465	2.41040	0.07651	-0.08246	0.02563	0.02203	0.01883
Ubiquitin	0.57	0.02498	0.16269	-0.12833	-0.34009	0.14417	0.10182	0.22777	0.11253	0.59464	6.03918	0.39674	-0.16048	-0.75384	0.01366	0.00000
Chymotrypsinogen	0.47	0.02974	-0.00659	-0.06535	-0.30858	0.08184	0.09151	0.20199	0.07274	0.26538	2.98656	0.00880	-0.08670	-0.59413	0.00805	0.00000
Ribonuclease A	0.38	0.03486	-0.25553	0.10459	-0.45477	-0.01001	0.08624	0.11261	-0.01467	0.01487	0.74316	-0.00613	-0.06106	0.04370	0.06285	0.01763
Trypsin	0.27	0.02679	-0.00622	-0.06330	-0.25571	0.06670	0.06826	0.13960	0.05272	0.18607	2.89050	0.06012	-0.08863	-0.33468	0.00668	0.00000

Table S9. Linear fit of aromaphilicity and hydrophobicity with sequential omission of proteins from data analysis.

Leave-out Protein	RMSE	R^2	Predicted Binding, M	Actual Binding, M	Delta, M
none	0.062	0.984	none	none	none
lysozyme	0.062	0.956	1.19	1.32	0.13
papain	0.055	0.991	0.91	0.82	-0.09
ubiquitin	0.046	0.994	0.43	0.57	0.14
α-Chymotrypsinogen A	0.074	0.985	0.50	0.47	-0.03
ribonuclease A	0.033	0.997	0.17	0.38	0.21
trypsin	0.069	0.984	0.33	0.27	-0.06
